# Principles of Intelligence: On Evolutionary Logic of the Brain

**DOI:** 10.3389/fnsys.2015.00186

**Published:** 2016-02-03

**Authors:** Joe Z. Tsien

**Affiliations:** ^1^Brain and Behavior Discovery Institute, Medical College of Georgia at Augusta UniversityAugusta, GA, USA; ^2^Banna Biomedical Research Institute, Brain Decoding Project ConsortiumBanna, China

**Keywords:** theory of connectivity, brain evolution, origin of intelligence, cell assembly, computational logic, neural cliques, functional connectivity motif, artificial intelligence

## Abstract

Humans and animals may encounter numerous events, objects, scenes, foods and countless social interactions in a lifetime. This means that the brain is constructed by evolution to deal with uncertainties and various possibilities. What is the architectural abstraction of intelligence that enables the brain to discover various possible patterns and knowledge about complex, evolving worlds? Here, I discuss the Theory of Connectivity–a “*power-of-two*” based, operational principle that can serve as a unified *wiring and computational logic* for organizing and constructing cell assemblies into the microcircuit-level building block, termed as *functional connectivity motif* (FCM). Defined by the *power-of-two* based equation, *N* = 2^*i*^−1, each FCM consists of the principal projection neuron cliques (*N*), ranging from those specific cliques receiving specific information inputs (*i*) to those general and sub-general cliques receiving various combinatorial convergent inputs. As the evolutionarily conserved logic, its validation requires experimental demonstrations of the following three major properties: (1) *Anatomical prevalence—*FCMs are prevalent across neural circuits, regardless of gross anatomical shapes; (2) *Species conservancy—*FCMs are conserved across different animal species; and (3) *Cognitive universality—*FCMs serve as a universal computational logic at the cell assembly level for processing a variety of cognitive experiences and flexible behaviors. More importantly, this Theory of Connectivity further predicts that the specific-to-general combinatorial connectivity pattern within FCMs should be preconfigured by evolution, and emerge innately from development as the brain’s computational primitives. This proposed design-principle can also explain the general purpose of the layered cortex and serves as its core computational algorithm.

Some of us may recall what Jeff Hawkins once told a Silicon Valley audience: “We don’t want to solve vision, we don’t want to solve language. We want to solve something in the brain that is more fundamental.” (Hawkins, 2004). What could be more fundamental to our understanding of the brain than the fascinating fields of vision, smell, touch, hearing, emotion, learning and memory, decision-making and motor control? The answers may find their roots in the Einsteinian quest for unifying principles in science (Adolphs, 2015).

Different animals can exhibit a drastically different sensory apparatus—such as electroreception (in electric eels and honeybees), magnetoception (in homing pigeons and mole rats), sonar (in bats and dolphins) or infrared detectors (in snakes and bed bugs). As such, different animals clearly construct very different models and perceptions in their brains about the worlds. Moreover, motor skills are also widely different—from digging beneath the dirt, swimming in the ocean, walking on the surface of the Earth or flying high in the sky. Therefore, the central mission of intelligence is to solve various problems in their natural and social environments in order to survive and thrive. This means that intelligence is ultimately about the ability to self-discover knowledge and patterns from a world full of uncertainties and infinite possibilities. If so, what is the wiring and computational logic that evolution should use to construct the brains? 

Throughout history, attempts to understand how the brain works have been frequently made via comparing the brain with various machines of that particular time—from pumps and engines to calculators and computers (von Neumann, 1958). Current comparisons between the brain and computers are often illustrated by a set of striking numbers; for example, the three-pound human brain, which consumes only 20 watts, vs. large supercomputers, which occupy an entire floor and burn tens of thousands of watts of electricity (Merolla et al., 2014). However, such superficial comparisons simply miss the point. The fundamental contrast between the brain and a computer lies in their design principles. These two devices have two completely different missions; computers are designed and programmed to perform well-defined, specific tasks with maximal speed and energy efficiency, whereas the brains have evolved to generate knowledge and adaptive behaviors. Thus, the fundamental question posed to neuroscientists and engineers alike is: how does the brain achieve this amazing feat?

Biologists have approached the question by using what is known as “the disassembling approach.” Roman Y. Cajal pioneered this approach one century ago, revealing the basic structural elements of the brain—such as neurons, axons, dendrites and synapses (Cajal, 1909, 1910; DeFelipe and Jones, 1988). Over the past few decades, a wealth of knowledge has been collected on many component parts of the brain from molecular to behavioral levels—ranging from the identification of neurotransmitters, ion channels and receptors (Noda et al., 1984; Seeburg et al., 1990; Monyer et al., 1992; Carlsson, 1993), elucidations of sensory detectors and cortical columns (Mountcastle, 1957; Hubel and Wiesel, 1962; Buck and Axel, 1991), the discovery of synaptic machineries and plasticity (Bliss and Collingridge, 1993; Frey and Frey, 2008; Südhof, 2012), the genetic manipulation or decoding of specific circuits (Tsien et al., 1996; Zemelman et al., 2002; Boyden et al., 2005; Zhang et al., 2013), and the enhancement of cognitive behaviors (Tang et al., 1999; Wang et al., 2009). Yet, this relentless push—or downward spiral—into ever finer details has created its own attraction—or black holes—from which too many of us may find too hard to resist intellectually and professionally.

Physicists, on the other hand, tend to use a very different approach; that is, they tend to first come up with a theory to describe the general principle of physical worlds using mathematics. Perhaps this is out of necessity, because the mysterious objects physicists study—such as the solar system and universes—are usually intangible. Their reliance on thought-experiments has served physics extremely well. Can we borrow this trick from physicists and perform some thought experiments?

## Power-of-Two Based Logic for Coping with Uncertainties and infinite Possibilities

Theoretical physicist Michio Kaku has pointed out that there are so many people who have worked so hard for so long, the neuroscientists have hardly come up with any theory about the design principles of intelligence (Kaku, 2014). Not necessarily agreeing with his conclusion, but I think that Dr. Kaku’s point should be well taken. Here, I would like to take the liberty to explore and discuss a *mathematical* approach to the following three questions: (1) What is the architectural abstraction principle for evolution to build the brain?; (2) How do neural networks give rise to intelligence that is capable of dealing with uncertainties and infinite possibilities, subsequently discovering knowledge and generating adaptive skills?; and (3) Is there a common mathematical principle that may relate to both of these questions?

**Figure 1:**
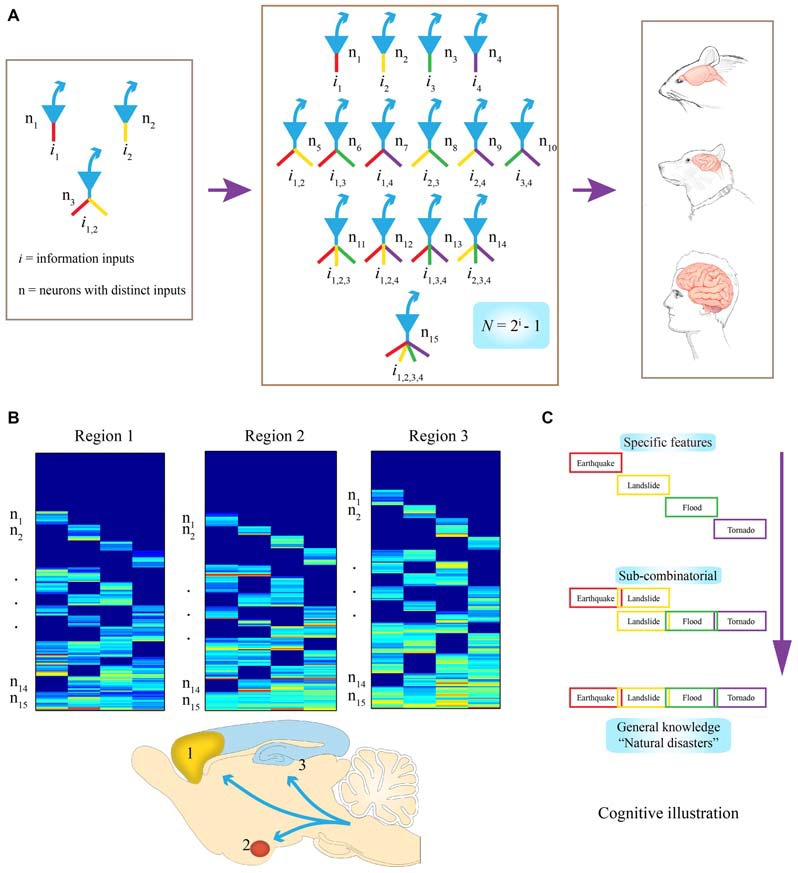
**Evolutionary logic guiding brain’s wiring and computation at the cell-assembly level. (A)** Examples of connectivity patterns within representative FCMs. On the left, a simple functional connectivity motif (FCM) with only three neurons for covering two distinct inputs (in red or yellow, *i*_1_ or *i*_2_, respectively). In the middle, a FCM consists of 15 distinct neural cliques (n_1–5_), which cover all possible connectivity patterns in order to process four distinct inputs (*i*_1–4_) by following the specific-to-general combinatorial wiring and computational logic. The proposed FCMs are predicted to be the cell-assembly level building blocks for constructing various brains (e.g., mouse, dog, and human are illustrated here). **(B)** Universality of this computational logic, which can be detected by measuring neural activation patterns in the form of a “bar-code”. Warm color bars illustrate the activations of these cell cliques. Schematic “bar-code” illustration of specific-to-combinatorial input connectivity motifs are predicted to be present in a wide range of brain regions regardless of anatomical shapes. Three bar codes all contain FCMs for processing four distinct inputs (*i* = 4), each consists of 15 distinct neural cliques (n_1–15_). **(C)** On the right, a “cognitive example” is provided for how the bar code-like activation patterns of FCMs give rise to a specific-to-general feature-extraction assembly that encodes specific features or memories, as well as various relational or generalized knowledge about four distinct fearful events. This specific-to-general computational logic can also be used to generate categorical motor behaviors.

If the ability to discover specific features and generalized knowledge from the complex, ever-changing worlds is the core function of the brain, the search for the brain’s design logic, I believe, can then be reduced to the question of how neurons should be wired to intelligently discover and organize various possible patterns. Recently, I put forth a “*power-of-two*” based Theory of Connectivity to explain how evolution and development might construct cell assemblies in such a way that would inherently cover all possible information (Tsien, 2015). At its core, I postulate the cell assemblies are not random, but rather should conform to the *power-of-two* based equation, *N* = 2^i^−1, to form the pre-configured building block termed as the functional connectivity motif (FCM). Instead of using a single neuron as the computational unit in some extremely simple brains, I denote that in the most of the brains, a group of neurons exhibiting the similar tuning properties, termed as a neural clique, should serve as the basic computing unit. This allows the system to avoid a catastrophic failure in the event of losing a single neuron (in engineering, the term for this phenomenon is “graceful degradation” or, simply, “robustness”). Here, *N* is the number of distinct neural cliques connected in different possible ways; *i* is the information types this FCM is dealing with. According to this equation, each FCM is predicted to consist of a full range of neural cliques that extract and process a variety of inputs in a combinatorial manner (Figure 1). Thus, depending on how many distinct information input a microcircuit is designed to handle, this equation, *N* = 2^i^−1, defines how many neural cliques are needed for a particular FCM.

To illustrate what this equation means in evolutionary and neurobiological terms, let’s imagine that 500~650 million years ago, a simple animal organism had only two missions: to find foods and mates (information *i* = 2); then, three neurons would be needed at a central node (the brain) to present all possible relationships or features, (*N* =2^2^−1 = 3; Figure 1A, left subpanel). In this case, *N*#1 or #2 encodes for foods or mates, respectively, with the *N*#3 receiving convergent inputs from *i*_1_ and *i*_2_. And representing the presence of both food and mates (thereby serving as a command neuron for concept or motor behavior, such as *good* or *approaching*, respectively). Likewise, if *i* increases to 3 or 4, the corresponding *N* required to cover all possible relationships or combinations arranged from a set of specific units to sub-general units and general units will be 7 or 15, respectively (Figure 1A, middle subpanel). Therefore, through this *power-of-two* based mathematical logic, evolution constructs the specific-to-general neural clique assembly, or FCM, as its basic building block of various brains (Figure 1A, right subpanels). Such conserved FCM architecture enables the representation of a range of possible combinations of relational features that the network can extract from various information sources. In other words, by conforming to this mathematical principle of *N* = 2^i^−1, the specific-to-general neural clique assembly provides an efficient and flexible algorithmic framework for encoding specific events, as well as various relational knowledge or skills (Figure 1B). As the evolutionary conserved wiring and computational logic, the following three critical predictions should be fulfilled:

### Anatomical Prevalence

The proposed FCMs should be widely employed across the brain’s deep central circuits, regardless of their anatomical shapes and appearances (Figure 1B). It should be noted that while principal projection neurons are usually excitatory neurons using fast neurotransmitter (*i.e.*, pyramidal cells in the CA1), some GABAnergic interneuron types can also serve as projection neurons (e.g., medium spiny neurons in the striatum, PV interneurons in certain parts of the amygdala). For the purpose of theoretical abstraction, local inhibitory interneurons crucial for controlling the local microcircuit dynamics are not discussed here. This anatomical prevalence can be tested by the demonstration of FCM’s existence in the almond-shaped amygdala, the stratum of the CA1 region, or the multi-layered cortex.

### Species Conservancy

The proposed FCM should be observed across the brains of different species. In other words, the specific-to-general neural clique assembly-based computational logic should also be conserved across a variety of animal species, from simple invertebrate organisms to primates. The ratio distribution of specific neural cliques and sub-general or general cliques within each FCM is likely to vary among individual brains. But as a species, its overall specification and abstraction is ultimately selected by specific environments in which different animal species have lived and evolved. This species conservancy can be tested and verified in classical animal model organisms from worms to mice to primates (Roth and Dicke, 2012).

### Cognitive Universality

It is also predicted that this computational logic should be universal across distinct cognitive tasks, ranging from appetitive behavior to social interaction and fearful episodes. At the functional level, the specific input-responsive neurons should represent specific features or details about incoming stimuli or events, whereas sub-general and general (most convergent) input-processing neurons are situated to extract categorical or combinational relationship features and knowledge. In the primary or secondary sensory processing circuits, specific and sub-general cells may converge to produce a variety of complex-feature cells within its sensory modality. However, in high association cortices, combinations of different features are geared toward generating novel combinational relationships across modalities for describing abstract knowledge for categorical events, people, and/or actions. For a “cognitive” example, when a person encounters or witnesses an earthquake, landslide, flood or tornado, either separately, combinatorially, or together (*i* = 4), existence of all fifteen types of principal neural cliques in an emotional memory circuit can readily capture various specific and/or combinational relationships, ranging from neural representation for “earthquake, ” or “earthquake and landslide,” to “tornado with flood and landslide” or the general concept of “natural disasters” (Figure 1C). This specific-to-general bar-code logic (Figure 1B), implemented at the cell-assembly level, intrinsically enables the microcircuits to discover potentially all sorts of cognitively important patterns; consequently, giving rise to categorical knowledge at the macro-scale network level.

## Mathematical and Biological Boundary of the Biological Brains

This proposed *specific-to-general* brain logic conforms to a mathematical principle in the equation *N* = 2^*i*^−1. However, due to exponential growth in input numbers *i*, the cost (in terms of cell resources) can quickly become prohibitive. For instance, in order to cover all possible patterns for processing 2, 3, 4, 10, 20, 30, 40 distinct perceptual inputs, an FCM would require 3, 7, 15, 1023, 1 048 675, 1 073 741 823, 1 099 511 627 775 neurons, respectively. Even the human brain, which is estimated to have 8.6 × 10^10^ neurons, can quickly run out of cells to be able to afford this exponential coverage. The best and necessary solution is to employ modular approaches, or a divide-and-conquer strategy, to segregate or stream information inputs through distinct sensory domains or submodular pathways.

For example, if a central node in a small neural circuit (e.g., *C. elegans*) needs to cover all possible connectivity wiring patterns to represent eight distinct types of inputs or information, a total of 255 principal projection neurons would be required (*N* = 2^8^−1 = 255) for this node. However, when a sub-modular approach is employed (e.g., dividing into a set of four inputs per subnode), the same 255 principal neuron sets can increase its processing capacity by a factor of 17 times (255 total cells/15 cells per sub-node = 17). Similarly, if a subnode or FCM was structured to process only three distinct information (*N* = 2^3^−1 = 7), 255 neurons can be used for 36 assemblies. Through evolution, *i* number should have been selected and confined by the complexity of given environmental demands in which organisms have lived for generations. This means that evolution has been forced by this mathematical cost-and-benefit analysis to use neuron resources efficiently and wisely, as evident from the evolutionarily conserved specific sensory pathways and cortical modalities. With more neurons available to the more complex animals, bigger *i* numbers (that their microcircuits can handle) would become, thereby leading to greater intelligence.

## The Cortex as the Evolutionary Scale-Up Solution for Large-Scale Computing

In order to extract an increasing amount of relational patterns across distinct sensory modalities, scaling up this *power-of-two* based microarchitecture is necessary, but can be a major challenge from an engineering perspective. I propose that the classic three- or six-layered cortex is the evolutionary solution to execute the FCM logic in a replicable large-scale fashion, as the brain evolves from small-scale circuits to larger circuits. In other words, input cortical layers should consist of most of the specific cliques and simple sub-general cliques (e.g., 2-event combinatorial cliques); whereas output layers would host most of the more complex sub-general cliques and general cliques (e.g., 3-event or 4 event-combinatorial cells). In fact, this is in general agreement that layer 4 neurons are simple cells and predominantly project to layers 2 and 3 from which neurons then descend to layers 5 and 6 (Benshalom and White, 1986; Peters and Payne, 1993; Bruno and Sakmann, 2006; Kaneko, 2013). Although a majority of these output cell cliques should be sub-general and general cliques, one should also expect a certain percentage of cells in these layers to be specific due to direct input from layer 4 (Constantinople and Bruno, 2013). It should be noted that layers 2/3 are also the output layers to other cortical regions (Ueta et al., 2013; Barbas, 2015). As a whole, this arrangement can enable the discovery and broadcasting of general and combinatorial patterns in the output layers while still being capable of retaining the ability for pattern discrimination.

## Different Logic for Neural Modulatory Systems

While the proposed FCMs are predicted to be prevalent across regions, I would like to suggest that evolution may use a rather distinct wiring logic to deal with neural modulatory circuits consisting of slow neurotransmitter-containing neurons. These neural modulatory circuits, such as the dopamine (DA) neuron circuit, are designed to add values, saliency, motivational and/or emotional features (Carlsson, 1993; Ferguson et al., 2002; Matsumoto and Hikosaka, 2009; Lammel et al., 2011; Wang and Tsien, 2011; Everitt and Robbins, 2015) onto the information patterns discovered by the fast neurotransmitter neurons. By adding various “flavors” to FCMs, these modulatory systems modulate the tuning properties, excitability, long-term stability, and temporal dynamics of neural cliques during learning, consolidation, retrieval, or imagination (Frey et al., 1990; Tsien et al., 2013).

## How Can This Theory of Connectivity Be Tested?

**Figure 2:**
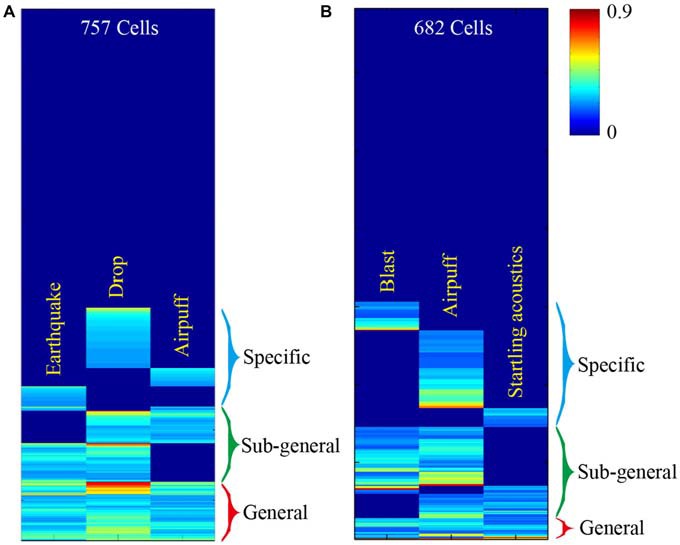
**Illustration of experiments to test this neural clique assembly-based computational logic. (A)** Specific-to-general neural cliques in the mouse CA1 region. A total of 757 CA units from five mice (*n* = 189 ± 29) were pooled together to generate this hierarchical clustering plot. Some cells responded to all three fearful stimuli (general clique), while some cell cliques (specific or sub-general cells) exhibited specific or sub-combinatorial selectivity to one or two types of stimulus, respectively. **(B)** Specific-to-general neural cliques in the mouse anterior cingulate cortex (ACC) region in responding three distinct fearful events. A total of 682 ACC units from six mice (*n* = 137 ± 43) were pooled together to generate this hierarchical clustering plot. The distinct fearful events are labeled. The CA1 and ACC figures were adopted from Lin et al. (2005) and Xie et al. (2013), respectively. The color scale bar indicates the *Z*-score normalized magnitude in firing changes within 2 s after stimulus onset.

This basic logic should be tested vigorously in a variety of experiments. For starters, large-scale neural recording techniques can be used to measure response specificity and convergence of principal neurons in the central circuits, while animals are subjected to fearful or appetitive stimuli without any prior training (Lin et al., 2005; Chen et al., 2009; Tsien et al., 2013; Guven-Ozkan and Davis, 2014). Our previous large-scale recording experiments examined how CA1 cells would respond to three distinct fearful events; namely, earthquakes, free-fall drops, and air-puffs (Lin et al., 2005, 2006). We found that the CA1 generated seven distinct major neural clique activation patterns in response to these three fearful episodes (Figure 2A). Similarly, we recorded from the mouse anterior cingulate cortex (ACC) while subjecting the mice to mild bomb blast, air puff, or acoustic startle (Xie et al., 2013). We again observed seven combinatorial neural cliques, arranged in a specific-to-general manner (Figure 2B).

At face value, the results from these large-scale *in vivo* recording experiments seem to be consistent with the proposed logic. However, the major caveat is that the units listed in these hierarchical cluster plots contained all of the recorded units, including putative pyramidal cells and local interneurons. Therefore, a critical test is to demonstrate this specific-to-general neural clique pattern in pyramidal cells. A convincing evidence would be to show the existence of seven types of pyramidal-cell cliques not only from the pooled datasets, but also from a single mouse dataset. Finally, to further examine the power-of-two based combinatorial wiring, one need to specifically design new experiments by increasing the fearful events from the previous three types to four types, and can still find all 15 distinct types of cliques in these regions. In addition, simple organisms such as worms, flies, and zebra fish may also offer unique advantages to test the proposed logic (Larsch et al., 2013; Guven-Ozkan and Davis, 2014; Kato et al., 2015; Wolf et al., 2015).

In summary, I propose that the principle of intelligence is rooted in a mathematical principle for guiding the brain design by evolution. Through evolution and development, cell-assembly connectivity in the unlearnt microcircuits should be already preconfigured and genetically programmed by this logic, which enables the brain, at its microcircuit level, to discover knowledge and generate flexible behaviors. This FCM logic also explains the general purpose and core computational algorithm behind the layered cortex. This design principle can be examined by developmental experiments, and modeled by neuromorphic engineers and computer scientists. However, it is important to note that artificial general intelligence based on the brain principles can come with great benefits and potentially even greater risks.

## Author Contributions

JZT is the sole author and wrote this Perspective article.

## Conflict of Interest Statement

The author declares that the research was conducted in the absence of any commercial or financial relationships that could be construed as a potential conflict of interest. The reviewer Dr. Tianming Liu declares that, despite having a past co-authorship with the author, Dr. Joe Z. Tsien, the review process was handled objectively and no conflict of interest exists.
